# Tribochemical Interactions between Graphene and ZDDP in Friction Tests for Uncoated and W-DLC-Coated HS6-5-2C Steel

**DOI:** 10.3390/ma14133529

**Published:** 2021-06-24

**Authors:** Joanna Kowalczyk, Monika Madej, Wojciech Dzięgielewski, Andrzej Kulczycki, Magdalena Żółty, Dariusz Ozimina

**Affiliations:** 1 Faculty of Mechatronics and Mechanical Engineering, Kielce University of Technology, 25-614 Kielce, Poland; mmadej@tu.kielce.pl (M.M.); ozimina@tu.kielce.pl (D.O.); 2 Air Force Institute of Technology, 01-494 Warsaw, Poland; wojciech.dziegielewski@itwl.pl (W.D.); andrzej.kulczycki@itwl.pl (A.K.); 3 Oil and Gas Institute-National Research Institute, 31-503 Cracow, Poland; magadalena.zolty@inig.pl

**Keywords:** graphene, diamond-like carbon, zinc dialkyldithiophosphate, lubricant additive, surface layers of solid elements

## Abstract

If a lubricant contains structures capable of conducting energy, reactions involving zinc dialkyldithiophosphate (ZDDP) may take place both very close to and away from the solid surfaces, with this indicating that ZDDP can be a highly effective anti-wear (AW) additive. The central thesis of this article is that the tribocatalytic effect is observed only when the energy emitted by the solids is transmitted by ordered molecular structures present in the lubricant, e.g., graphene. The friction tests were carried out for 100Cr6 steel balls in a sliding contact with uncoated or W-DLC-coated HS6-5-2C steel discs in the presence of polyalphaolefin 8 (PAO 8) as the lubricant, which was enhanced with graphene and/or ZDDP. There is sufficient evidence of the interactions occurring between ZDDP and graphene and their effects on the tribological performance of the system. It was also found that the higher the concentration of zinc in the wear area, the lower the wear. This was probably due to the energy transfer resulting from the catalytic decomposition of ZDDP molecules. Graphene, playing the role of the catalyst, contributed to that energy transfer.

## 1. Introduction

Friction is a phenomenon with either negative or positive effects. When undesirable, it needs to be reduced. Researchers all over the world are looking for ways to achieve this. One of the basic methods to handle this problem, especially in automotive and mechanical systems, is by applying lubricants [1]. Lubricants help reduce friction between two moving metal surfaces, but their effectiveness can be improved by properly selecting the additives [1,2].

One of the most common lubricant additives is zinc dialkyldithiophosphate, ZDDP, added, for instance, to engine oils. Originally, in 1940 [3], it was developed as an antioxidant and detergent [4]; its other potential applications were suggested later [3]. In the literature, ZDDP is classified as an antiwear and anticorrosion additive [5]. The presence of ZDDP contributes to the formation of protective lubricating films [4]. In the case of ZDDP, however, the lubrication mechanism is quite complicated because there are three [6,7] interactions with the active elements [6,7,8], i.e., zinc, phosphorus and sulfur. Water and oxygen are also active, but their presence increases the complexity of the lubrication mechanism. The composition of the film forming on a solid surface is dependent on the operating conditions, type of contact between the moving parts, as well as the length of its aliphatic chains [6,7].

Lubricant additives containing phosphorus and sulfur are reported to be effective as extreme pressure and antiwear (EP–AW) additives. The proposed mechanism of operation involves tribofragmentation, during which an additive is split into highly reactive fragments, reacting with an uncoated (unprotected) metal surface [9].

In the case of phosphorus- and sulfur-containing oils, the layer that forms on the solid surface is rich in phosphorus when the load is moderate; at higher loads it is rich in sulfur.

Numerous studies confirm that ZDDPs are efficient as lubricant additives. Much progress has been made in the area of elemental analysis of the films formed. Martin et al. [10,11] and Bell et al. [12,13] describe the formation of a layer of vitreous polyphosphorus about 500 nm in thickness in the presence of ZDDP, above which there is an approximately 100 nm thick organic layer. They also report the occurrence of iron sulfide between the phosphorus layer and the iron-based substrate, showing an adsorptive or antiwear function. The zinc compound is considered to be the best antiwear inhibitor of all metals studied [14].

Phosphorus has good EP and AW properties when added to base oils [5]. Compared with sulfur, phosphorus is a better antiwear agent [5,15]. There has been much national and international research into this additive [16]. Investigations by Luo et al. [17] concerning the tribological properties of an aqueous solution of nonylphenol polyoxyethylene ether phosphate ester (PPE) [17] show that it is possible to reduce the coefficient of friction to 0.15 and wear even up to 50%. Concentrations higher than 0.5% by weight guarantee that the lubricant is likely to satisfy both the lubricating and antiwear requirements. Ren et al. [18] consider the use of a P-N-type ionic liquid in synthetic ester (PETO) and water-based emulsion and they indicate that the tribological response of the materials in contact is better in the presence of oligomeric phosphorus. Wu et al. [19] analyze the performance of three different triazine compounds added to water-glycol fluids to act as water-soluble antiwear additives. The results show that the compounds provide higher resistance to extreme pressure and wear [16].

The tribological tests concerning the performance of polyalphaolefin 8 (PAO 8) with and without ZDDP and carbon nanotubes (CNTs) [20] show clear interactions between the two additives. The addition of ZDDP resulted in lower linear wear on uncoated steel discs but higher linear wear when these were coated with hydrogenated amorphous carbon (a-C:H). Amorphous carbon or diamond-like carbon (DLC) has properties similar to crystalline diamond, which vary depending on the bonds between carbon atoms [21]. DLC coatings can be classified according to a number of different criteria. When the presence of foreign elements in the structure is considered, DLC coatings fall into doped and undoped. The former can be subdivided into metal-doped and nonmetal-doped, with metals and non-metals including Ti, W, Mo, Cr, and Si, F, N, respectively [22].

The development of nanotechnology, which enabled the manufacture of advanced materials, led to the development of lubricants with better properties, which was achieved by introducing antiwear additives in the form of nanomaterials. Carbon nanomaterials have good lubricating properties, hence their common use as additives. Carbon-based nanomaterials include carbon nanotubes, fullerenes and graphene [1]. Nanotubes with their varied morphology can have different mechanical, electronic, chemical and magnetic properties [23].

Graphene is a two-dimensional carbon allotrope, characterized by good mechanical, electrical and thermal properties, which is why it is used in various applications, including engineering, chemistry and physics [24,25,26]. Additionally, carbon atoms in graphene bond easily, which makes it an effective additive, reducing friction in mechanical systems, e.g., automotive engines.

Zhang et al. [27] used graphene oxides altered with oleic acid to create graphene nanosheets; they obtained a lubricant suitable for antiwear applications. Azman et al. [28], on the other hand, mixed graphene with PAO 10 synthetic oil (95 vol%) and palm oil based trimethylolpropane ester (15 vol% of TMP), which reduced the wear scar size by 15% [1].

Saurín et al. [29] experimented with 1–2- and 1–10-layer graphene dispersed in ionic liquid at a concentration of 0.1% by weight. The lubricant variants were tested in a system with polymer-steel or ceramic-steel contact surfaces. The ionic liquid containing 1–10-layer graphene was reported to reduce the coefficient of friction and the wear rate.

Garcia et al. [30] compared the performance of different lubricants enhanced with graphene. The study was conducted for commercial PAO 6 and ‘green’ choline chloride (ChCl)-based deep eutectic solvents combined with urea, ethylene glycol (EG) or malic acid. A steel–steel contact was analyzed using a tribometer with a block-on-ring configuration. The lowest coefficient of friction was observed for ChCl-EG + graphene, which is biodegradable.

The objective of this study was to determine whether graphene and ZDDP, added separately or together, would reduce the coefficient of friction and linear wear. It was a novelty to use graphene as an additive taking part in the tribocatalytic reaction with the solid surfaces and ZDDP as a friction modifier additive. The friction tests conducted at relatively low loads showed that, like other structured carbon forms, especially carbon nanotubes (CNTs), graphene enhanced the effectiveness of ZDDP as an antiwear additive. That, however, differed depending on the structure and properties of the materials in contact.

## 2. Experiment

The tribological tests were carried out using a TRB^3^ tribometer. The ball-on-disc configuration operated at a humidity of 37 ± 15% and an ambient temperature (T_0_) of 24 ± 4 °C. The test parameters were as follows: load 10 N, sliding speed 0.1 m/s and sliding distance 1000 m.

Table 1 shows the four lubricants used in the friction tests. It is important to mention that no dispersing agents were introduced to prevent the interaction between the additives.

The testing was performed using polyalphaolefin 8 (PAO 8) as the base oil (Table 2). The hydrocarbon structure of the oil is a result of catalytic oligomerization of linear α-olefins having from 8 to 12 carbon atoms in the chain. Table 2 illustrates the basic properties of polyalphaolefin 8.

ZDDP was introduced to act as the friction modifier additive. The concentration of ZDDP used as the AW/EP additive in commercial applications (engine oils, hydraulic fluids or transmission fluids) remains at about 2 mmol/kg. From the authors’ earlier research as well as a review of the literature on the subject, e.g., [31] it is clear that dimers predominate at concentrations higher than approx. 15 mmol/kg, with this affecting the adsorption and chemisorption on the contact surfaces. As the investigations focused on the interaction between ZDDP and graphene in friction at a relatively low load, ZDDP was added to the different lubricants at appropriate concentrations so that dimers would be the prevalent form. Thus, ZDDP was introduced at a concentration of 1.5% (*m*/*m*), which corresponds to about 20 mmol/kg.

The concentration selected for graphene was 0.005% (*m*/*m*); it was equal to that of carbon nanostructures studied by the authors earlier [20].

The lubricants were prepared by dispersing graphene and ZDDP ultrasonically.

Table 3 shows the properties of PAO 8 after ZDDP was added.

Graphene flakes with a very low oxidation level produced by Advanced Graphene Products were used in the tests. Table 4 shows the main properties of graphene.

HS6-5-2C high-speed steel with and without a W-DLC coating was used in the experiments. The composition of HS6-5-2C steel is given in Table 5.

To obtain high hardness of about 65 HRC, the steel can be subjected to austenitization at a temperature of 1150 °C, which is followed by tempering at 560 °C [32].

The system consisted of a ball made of 100Cr6 steel and a disc made of HS6-5-2C steel. Uncoated as well as W-DLC coated discs were used to compare the lubricant behavior under different friction conditions.

**Figure 1:**
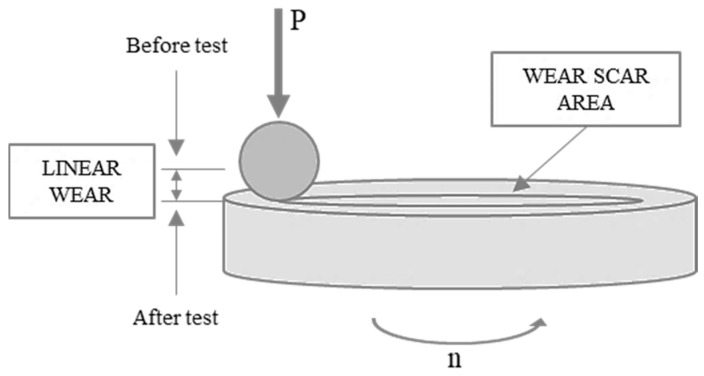
Tribological measurements.

Diamond-like carbon (DLC) coatings are very popular because they are characterized by good tribological and anticorrosion properties as well as high hardness [17,18]. The W-DLC coating was 3.5 μm in thickness. As shown in Figure 1, the tribological tests involved measuring: the coefficient of friction, linear wear and the wear scar surface area.

The values measured with two friction sensors mounted on the TRB^3^ tribometer were saved automatically. The software then calculated the coefficient of friction from the friction force, dividing it by the applied load, P = 10 N. Each tribological test was repeated three times. The wear scar area was studied by means of a Leica DCM8 confocal microscope operating in the interferometry mode. The linear wear was measured as the depth to which the ball had removed the material on the disc surface.

The elemental constituents of the contact surfaces were determined at the start of each test and after it was completed using a Phenom XL scanning electron microscope, fitted with an EDS detector for analyzing the chemical composition of materials.

## 3. Results

### 3.1. a-C:H Coating

**Figure 2:**
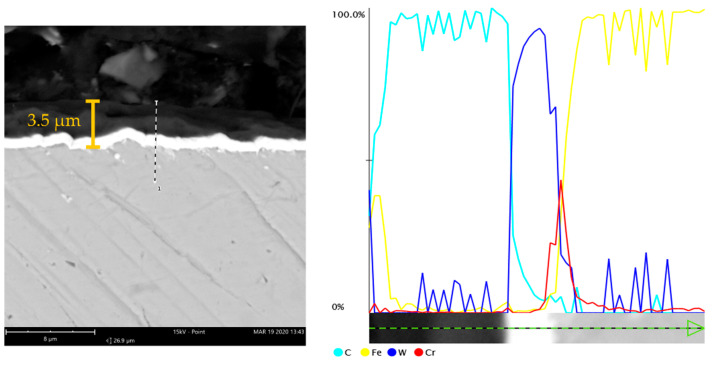
SEM microphotograph and results of the EDS line profile analysis of the W-DLC coating.

Figure 2 presents the cross-section of the W-DLC coating and a thickness measurement of 3.5 μm.

### 3.2. Tribological Tests

**Figure 3:**
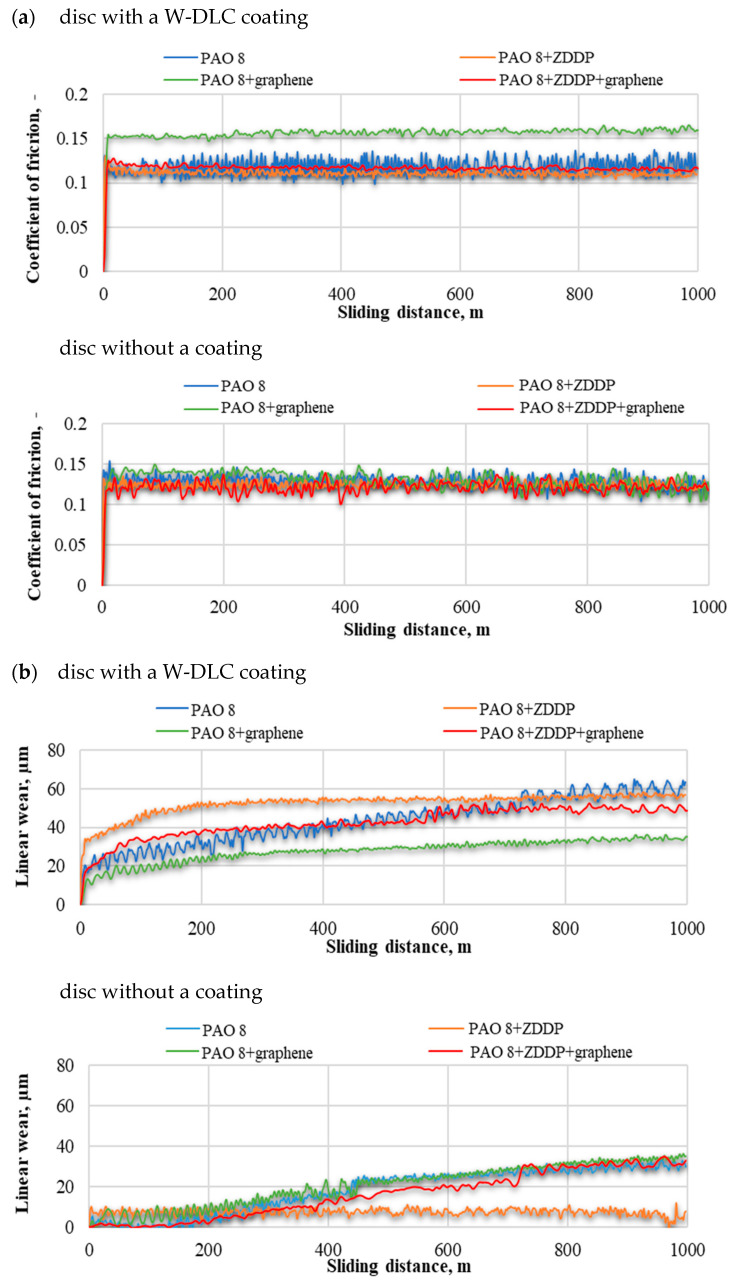
The tribological parameters: (**a**) coefficient of friction and (**b**) linear wear.

Figure 3 illustrates the tribological properties of all the systems tested. The performance of PAO 8 and PAO 8 + ZDDP with and without the addition of graphene is also depicted [20].

From the test results, it is clear that the lowest coefficient of friction was reported for the coated discs lubricated with PAO 8 + ZDDP. The results concerning the uncoated discs indicate that the coefficient of friction was lower when lubrication was provided by: PAO 8, PAO 8 + ZDDP or PAO 8 + ZDDP + graphene. The highest value of the coefficient of friction for both the coated and uncoated discs was obtained in the presence of PAO 8 + graphene. The linear wear, however, reaches the lowest value for PAO 8 + graphene lubricating the coated discs and for PAO 8 + ZDDP applied on the uncoated discs. The highest value of linear wear was observed for the coated discs in the presence of PAO 8 and for the uncoated discs lubricated by PAO 8 + graphene.

**Figure 4:**
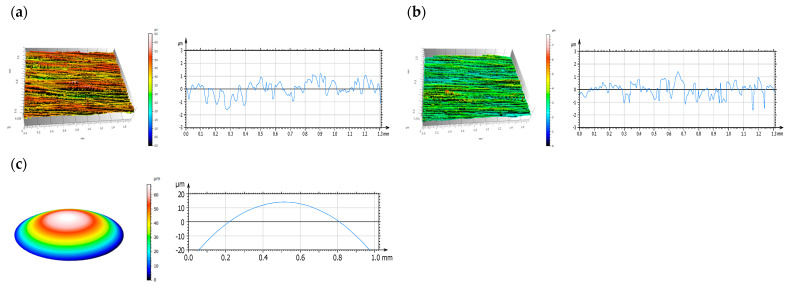
Surface topography: (**a**) the W-DLC coated HS6-5-2C steel disc, (**b**) the uncoated HS6-5-2C steel disc and (**c**) the 100Cr6 steel ball, before the tribological tests.

**Figure 5:**
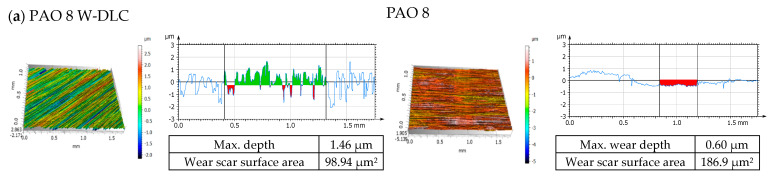

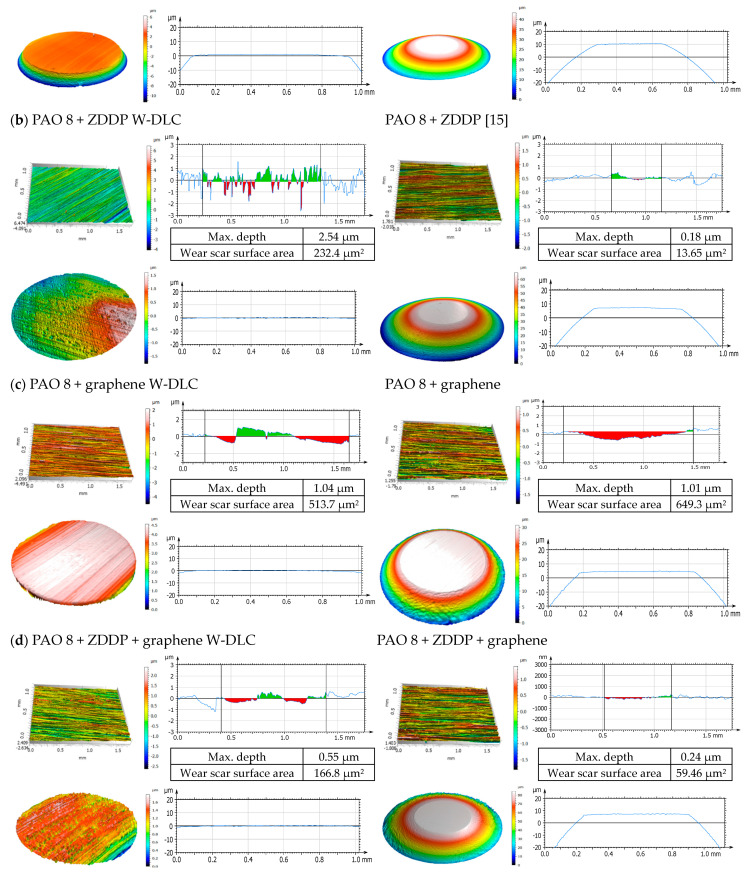
Isometric views and primary profiles of the discs and balls after the tribological tests: (**a**) PAO 8, (**b**) PAO 8 + ZDDP, (**c**) PAO 8 + graphene and (**d**) PAO 8 + ZDDP + graphene.

Figure 4 and Figure 5 show the results obtained with the confocal microscope operating in the interferometry mode. Figure 4 illustrates the values recorded prior to the tribological tests. As can be seen, W-DLC coatings contributed to lower peaks and shallower valleys than was the case of uncoated surfaces [20]. The isometric images on the left show the surface topographies along the height. The roughness profiles are on the right; the *x*-axis depicts the length of the sample in nm, and the y-axis illustrates the roughness height in µm.

Figure 5 provides the measurement values obtained for the discs and balls after the tribological tests [20]. On the left, there are the isometric images and, on the right, there are the roughness profiles.

The surface texture parameters obtained for the discs and balls before and after the tribological tests, shown in Table 6, are: Sa—arithmetic mean height, Sq—root mean square height, Sp—maximum peak height, Sv—maximum valley height, Sz—maximum height, Ssk—skewness and Sku—kurtosis.

**Figure 6:**
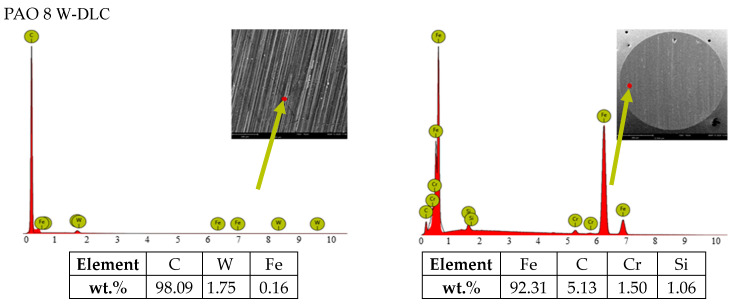

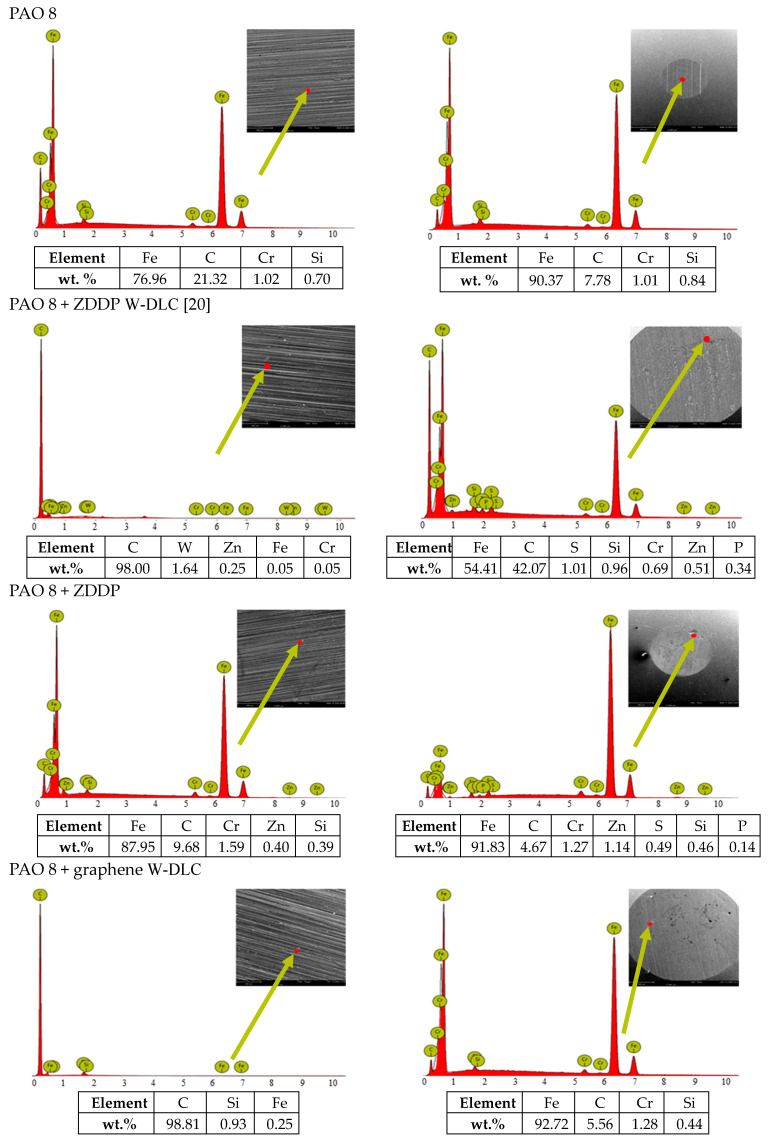

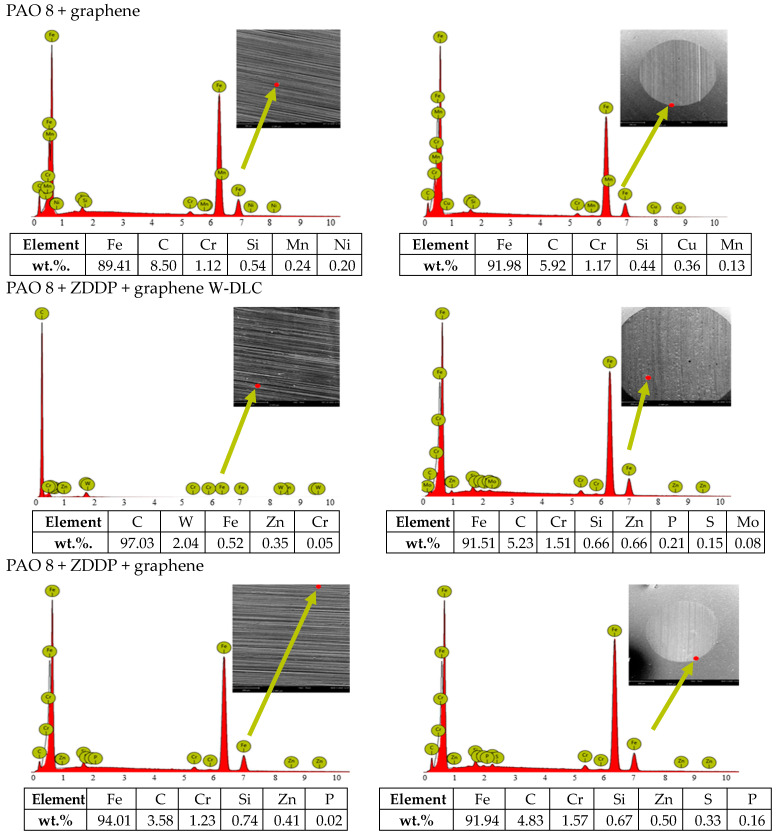
SEM images: surface morphologies and characteristic spectra (EDS) in the selected microareas along the wear tracks on the discs and balls after fiction contact.

Figure 6 depicts wear surface areas on the discs and balls after friction under lubricated conditions observed with a scanning electron microscope as well as EDS elemental analysis data obtained for selected microareas. The elemental analysis took into account the concentration of elements, depending on the type of disc and lubricant used. For the DLC-coated discs, the contents of such elements as C, W, Cr and Fe were studied, while for the uncoated steel discs, the contents of Fe, C, Cr and Si were measured. However, when lubricant was enhanced with ZDDP, the elements of interest were Zn, P and S.

## 4. Discussion

The experimental data shows that the tribological parameters were affected by the lubricant additives. The presence or absence of a coating on the disc surface proved to be significant, too.

From the chemical analysis of the W-DLC -coated disc, it is clear that the outer layer consists of carbon (Figure 2). The interlayer, however, is made up of two sublayers: tungsten and chromium. The interlayer improves the adhesion of the coating to the substrate; it modifies the transfer of energy from the solid material to the lubricant layer. There was interaction between W and carbon from W-DLC with formation of WC carbides. Through this tungsten diffuse to the surface [33].

The data in Figure 3a,b suggest that:

(a) uncoated HS6-5-2C steel
  - Coefficient of friction: For PAO + graphene, the coefficient was much higher than that obtained for the other lubricant variants.
  - Wear scar size: When ZDDP was added to PAO, the wear scar area and wear depth on the disc were greater. Similar observations were made for PAO + graphene. Lubricant containing both additives, i.e., ZDDP and graphene, caused wear much smaller in size, yet still its value was higher than that reported for PAO with no additives.
  - Linear wear: The addition of ZDDP to PAO resulted in lower values of linear wear. For PAO + graphene, they were even lower. The wear measured for PAO + ZDDP + graphene was similar to that obtained for PAO + ZDDP.
(b) W-DLC -coated HS6-5-2C steel
  - Coefficient of friction: There were hardly any differences in the values between the mixtures tested.
  - Wear scar size: The addition of ZDDP to PAO was responsible for a considerable decrease in the wear scar area and the wear depth on the disc. The values obtained for PAO + graphene were higher than those reported for PAO. Adding both ZDDP and graphene caused a significant decrease in the wear scar size; it was much smaller than that measured when PAO with no additives was used.
  - Linear wear: The tests carried out for PAO + ZDDP show that there was a substantial decline in linear wear. For the other lubricant variants, the values of this parameter were approximately the same.

From the above observations, it is evident that the W-DLC coating alters the behavior of the tribological system operating at low loads. Obviously, there is a strong relationship between the composition of the lubricant tested and the wear of the steel elements in contact, as the addition of ZDDP reduces wear. However, for a disc coated with DLC, the experimental results were different: ZDDP appeared to promote wear. A new finding is that the wear was very high on a disc coated with W-DLC as well as on an uncoated disc when the lubricant was PAO 8 + graphene. Similar observations were made for CNTs [20], but the effect was much smaller than for graphene. It can be concluded that both graphene and ZDDP contribute to the worsening of the surface properties of the W-DLC-coated steel disc and the uncoated steel ball, and consequently to their more intensive wear. This is particularly surprising for PAO 8 + graphene. Graphene can be seen as abrasive responsible for significant wear of the elements in contact. However, unlike ZDDP, it is not a chemically active substance reacting with the solid surface. Thus, the mechanism of intensive wear must be different. It is known that at low loads, friction modifier additives, particularly those becoming active when in contact with such materials as 100Cr6 steel (e.g., ZDDP), may react with the solid surface to weaken it, which will lead to higher wear. This mechanism does not apply when the disc is coated with W-DLC or when PAO 8 + graphene is used.

As indicated in [20], ordered carbon structures (CNTs, graphene) may be effective to remove the energy from the solid surface. This energy transfer may affect the bonding in the surface layer, which will cause the weakening of the surface layer, making it more susceptible to wear. This mechanism seems likely for the test conditions described here.

Additionally, there is a clear influence of graphene on the kinetics of the tribochemical reactions involving ZDDP. The differences in the concentrations of Zn, P and S accumulated on the contact surfaces (ball and disc) indicate that graphene modifies the reaction process. This effect is dependent on the structure of the surface layer of the disc being in sliding contact with the ball. For a disc coated with W-DLC, graphene catalyzes the molecular changes in ZDDP (higher concentration of Zn on both contact surfaces). In the case of a steel disc, graphene inhibits the reaction process (lower concentration of Zn on the disc surfaces and unchanged concentration of Zn on the ball surface).

The analysis of the surface topographies after the friction tests reveals that for the different lubricant variants, the surface texture of the disc is less varied (lower values of Sp, Sv and Sz) when the surface texture of the ball is more varied (higher lower values of Sp, Sv and Sz). The variability of results was greater for a system with an uncoated disc, which may suggest that the transfer of energy from the solids in contact and, consequently, the degradation of their surfaces cannot be fully prevented.

**Figure 7:**
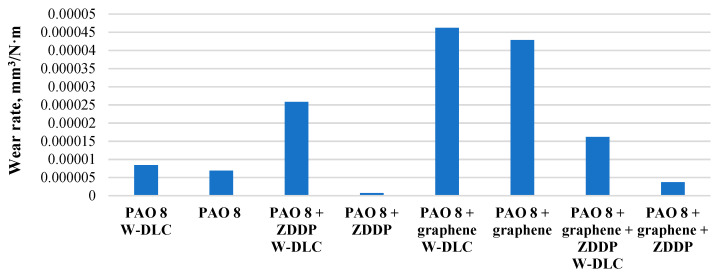
Wear rate of discs after tribological tests.

Figure 7 illustrates the wear rate of discs after tribological tests. The experimental data show that the lower wear rate was observed for uncoated disc when the lubricant was PAO 8 + ZDDP. The higher wear rate was noticed for coated disc after lubrication with the PAO 8 + graphene. For all cases, the lower wear rate was observed for the uncoated discs. After lubrication PAO 8 + graphene the wear rate was the higher for uncoated and coated disc. After adding the ZDDP to PAO 8 + graphene the wear rate has decreased drastically. For the disc with the a-C:H coating the lower wear rate was observed after lubrication PAO 8 + ZDDP + graphene than after lubrication PAO 8 + ZDDP. In this case, graphene reduced the wear.

**Figure 8:**
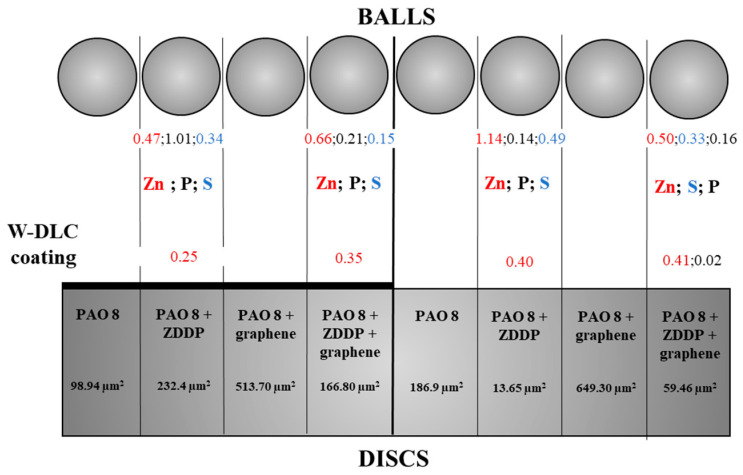
Concentrations of elements in the wear scar surface area, depending on the type of lubricant and the type of disc used.

From the data depicted in Figure 8, which correspond to those presented in Figure 3b, it is apparent that, for W-DLC -coated discs, the higher the concentration of Zn accumulated on the ball surface, the higher the linear wear on the disc surface. Additionally, conversely, if the concentration of Zn accumulated on the ball surface increases, there is a decrease in the linear wear on the disc.

It is difficult, however, to relate the concentration of accumulated Zn to the size of wear scars. Generally, when the disc was coated with W-DLC, the concentration of accumulated Zn was lower than that observed on an uncoated disc. Similar results were obtained for CNTs, as described in [20].

There is a clear difference in the concentrations of Zn, P and S accumulated on both contact surfaces between the systems studied, which resulted from the composition of the lubricant; the addition of graphene to PAO, separately or together with ZDDP, affected the system performance.

It was also found that the wear scar size was smaller at higher concentrations of elements occurring after friction, i.e., Zn, S and P, when the lubricant contained ZDDP. This was probably due to the transfer of energy.

The analysis of the interactions of ZDDP and graphene shows that graphene modifies the tribological reactions of ZDDP. ZDDP used as the antiwear additive undergoes physical adsorption on the lubricated surface as well as tribochemical reactions leading to the separation of Zn from the molecules containing P and S. According to the literature, the products of the ZDDP decomposition modify the surface structure of the materials in contact, preventing their wear. When tested tribologically, the additive (ZDDP) contributed to smaller wear scar sizes in a system with uncoated discs.

For DLC-coated discs, the linear wear was almost always higher (Figure 4b) than that observed for uncoated discs. A considerable increase in linear wear was reported in the presence of PAO 8 + ZDDP + graphene when W-DLC -coated discs were employed. Different results were obtained for steel discs without a W-DLC coating, when the use of ZDDP led to a reduction in linear wear.

## 5. Conclusions

The results of this study show that:

- The addition of ZDDP to the lubricant tested resulted in lower linear wear when the steel discs were uncoated.
- The use of ZDDP resulted in smaller wear scars when the disc had a W-DLC coating.
- Graphene added to the lubricant causes more intensive transport of energy from the solid to the system. This could be the reason why for PAO + graphene the wear was very high. In the presence of both additives, part of this energy reaches ZDDP molecules, which undergo triboreactions faster than when ZDDP is the only additive (higher concentrations of Zn, P and S). The tribological phenomena are greatly dependent on the surface properties of the solid elements in contact. The results obtained for W-DLC-coated steel discs differed from those reported for uncoated discs. It is thus evident that graphene and ZDDP interact, and the mechanism of this interaction is likely to be as follows. Graphene increases the transfer of energy from the solid surface to the molecules inside the lubricating film. Part of this energy is supplied to ZDDP molecules, initiating reactions leading to their decomposition. The rate of triboreactions involving ZDDP decreases, and so does the friction and wear of the contact surfaces (ball and disc).

The above hypothesis is consistent with the hypothesis presented in [20] but both should still be verified through further research.

## Figures and Tables

**Table 1:** Lubricants subjected to tribological testing.

Lubricant Composition	Symbol
PAO 8	PAO 8
PAO 8 + 1.5% ZDDP	PAO 8 + ZDDP
PAO 8 + 0.005% graphene	PAO 8 + graphene
PAO 8 + 1.5% ZDDP + 0.005% graphene	PAO 8 + ZDDP + graphene

**Table 2:** Properties of polyalphaolefin 8 (PAO 8).

Property	Value
Specific gravity (at 15.6 °C)	833 kg/m^3^
Kinematic viscosity at a given temperature [°C]:	
40	46.4 mm^2^/s
−40	19 570 mm^2^/s
Viscosity Index	138

**Table 3:** The basic properties of zinc dialkyldithiophosphate (ZDDP).

Property	Value
Density (at a temperature of 25 °C)	1160 kg/m^3^
Kinematic viscosity (at a temperature of 40 °C)	150 mm^2^/s
Content:	
Zn	9.0 wt%
P	8.5 wt%
S	16.5 wt%

**Table 4:** The basic properties of graphene.

Property	Value
Oxygen content	1–2%
Chemical composition–C	99.8%

**Table 5:** Chemical composition of HS6-5-2C high-speed steel.

Element	Percentage Composition
C	0.82–0.92
W	6–7
Mo	4.5–5.5
Cr	3.5–4.5
V	1.7–2.1
Co	≥0.5
Si	≥0.5
Mn	≥0.4
Ni	≥0.4
Cu	≥0.3
P	≥0.03
S	≥0.03

**Table 6:** Surface texture parameters for (**a**) the uncoated steel discs and (**b**) the W-DLC -coated steel discs in contact with the steel balls before and after the tribological tests.

**(a)**										
**Surface Texture Parameters**	**Before Test; Uncoated Disc**		**PAO 8**		**PAO 8 + ZDDP**		**PAO 8 + Graphene**		**PAO 8 + ZDDP +** **Graphene**	
	**Disc**	**Ball**	**Disc**	**Ball**	**Disc**	**Ball**	**Disc**	**Ball**	**Disc**	**Ball**
Sa (μm)	0.40	0.14	0.44	0.64	0.39	2.09	0.28	6.56	0.32	7.93
Sq (μm)	0.49	0.40	0.56	0.90	0.49	2.46	0.36	7.58	0.41	9.17
Sp (μm)	1.52	3.43	1.90	1.52	1.78	4.89	1.19	13.12	1.40	15.84
Sv (μm)	1.33	23.62	5.03	4.08	2.02	7.71	1.27	13.75	1.70	17.25
Sz (μm)	2.85	27.05	6.93	5.60	3.80	12.61	2.46	26.88	3.08	33.08
Ssk (-)	−0.17	−47.21	−0.32	−1.72	−0.24	−0.14	−0.36	−0.00	−0.51	−0.01
Sku (-)	2.39	2723	3.83	7.05	2.85	2.05	3.13	1.80	3.57	1.82
**(b)**										
Sa (μm)	0.39	0.14	0.64	0.36	0.56	0.48	0.47	0.26	0.38	0.10
Sq (μm)	0.50	0.40	0.82	0.47	0.74	0.59	0.61	0.33	0.51	0.12
Sp (μm)	1.60	3.43	2.87	3.95	6.47	1.60	1.68	3.65	2.08	0.16
Sv (μm)	2.11	23.62	3.70	5.32	4.09	1.78	2.99	1.10	2.65	0.39
Sz (μm)	3.71	27.05	6.56	9.27	10.57	3.38	4.67	1.49	4.73	0.55
Ssk (-)	−0.39	−47.21	−0.70	0.87	−0.91	0.54	−0.29	−1.13	−0.71	−0.81
Sku (-)	3.66	2723	3.54	5.50	5.07	2.61	3.85	3.65	5.02	3.04

## Data Availability

The data presented in this study are available on request from the corresponding author.
